# Biphasic voltage‐dependent inactivation of human Na_V_1.3, 1.6 and 1.7 Na^+^ channels expressed in rodent insulin‐secreting cells

**DOI:** 10.1113/JP275587

**Published:** 2018-03-30

**Authors:** Mahdieh Godazgar, Quan Zhang, Margarita V. Chibalina, Patrik Rorsman

**Affiliations:** ^1^ Oxford Centre for Diabetes, Endocrinology and Metabolism, Radcliffe Department of Medicine University of Oxford Churchill Hospital Oxford OX3 7LE UK; ^2^ Metabolic Physiology Department of Neuroscience and Physiology Medicinaregatan 11 Gothenburg S‐413 09 Sweden

**Keywords:** voltage‐gated sodium channels, pancreatic beta‐cell, voltage‐dependent inactivation, insulin secretion, electrical activity

## Abstract

**Key points:**

- Na^+^ current inactivation is biphasic in insulin‐secreting cells, proceeding with two voltage dependences that are half‐maximal at ∼−100 mV and −60 mV.
- Inactivation of voltage‐gated Na^+^ (Na_V_) channels occurs at ∼30 mV more negative voltages in insulin‐secreting Ins1 and primary β‐cells than in HEK, CHO or glucagon‐secreting αTC1‐6 cells.
- The difference in inactivation between Ins1 and non‐β‐cells persists in the inside‐out patch configuration, discounting an involvement of a diffusible factor.
- In Ins1 cells and primary β‐cells, but not in HEK cells, inactivation of a single Na_V_ subtype is biphasic and follows two voltage dependences separated by 30–40 mV.
- We propose that Na_V_ channels adopt different inactivation behaviours depending on the local membrane environment.

**Abstract:**

Pancreatic β‐cells are equipped with voltage‐gated Na^+^ channels that undergo biphasic voltage‐dependent steady‐state inactivation. A small Na^+^ current component (10–15%) inactivates over physiological membrane potentials and contributes to action potential firing. However, the major Na^+^ channel component is completely inactivated at −90 to −80 mV and is therefore inactive in the β‐cell. It has been proposed that the biphasic inactivation reflects the contribution of different Na_V_ α‐subunits. We tested this possibility by expression of TTX‐resistant variants of the Na_V_ subunits found in β‐cells (Na_V_1.3, Na_V_1.6 and Na_V_1.7) in insulin‐secreting Ins1 cells and in non‐β‐cells (including HEK and CHO cells). We found that all Na_V_ subunits inactivated at 20–30 mV more negative membrane potentials in Ins1 cells than in HEK or CHO cells. The more negative inactivation in Ins1 cells does not involve a diffusible intracellular factor because the difference between Ins1 and CHO persisted after excision of the membrane. Na_V_1.7 inactivated at 15‐­20 mV more negative membrane potentials than Na_V_1.3 and Na_V_1.6 in Ins1 cells but this small difference is insufficient to solely explain the biphasic inactivation in Ins1 cells. In Ins1 cells, but never in the other cell types, widely different components of Na_V_ inactivation (separated by 30 mV) were also observed following expression of a single type of Na_V_ α‐subunit. The more positive component exhibited a voltage dependence of inactivation similar to that found in HEK and CHO cells. We propose that biphasic Na_V_ inactivation in insulin‐secreting cells reflects insertion of channels in membrane domains that differ with regard to lipid and/or membrane protein composition.

## Key points

- Na^+^ current inactivation is biphasic in insulin‐secreting cells, proceeding with two voltage dependences that are half‐maximal at ∼−100 mV and −60 mV.
- Inactivation of voltage‐gated Na^+^ (Na_V_) channels occurs at ∼30 mV more negative voltages in insulin‐secreting Ins1 and primary β‐cells than in HEK, CHO or glucagon‐secreting αTC1‐6 cells.
- The difference in inactivation between Ins1 and non‐β‐cells persists in the inside‐out patch configuration, discounting an involvement of a diffusible factor.
- In Ins1 cells and primary β‐cells, but not in HEK cells, inactivation of a single Na_V_ subtype is biphasic and follows two voltage dependences separated by 30–40 mV.
- We propose that Na_V_ channels adopt different inactivation behaviours depending on the local membrane environment.

## Introduction

Voltage‐gated Na^+^ (Na_V_) channels are expressed in nearly all electrically excitable cells where they play a key role in action potential initiation and generation (Hille, 2001). Na_V_ channels exhibit a dual dependence on voltage: depolarization results in both rapid activation and a slower time‐dependent inactivation. During inactivation, the Na_V_ channels enter a non‐conducting state. Reversal of the ‘inactivated’ state requires hyperpolarization of the membrane, the extent of which can vary according to cell type and Na_V_ subtype (Catterall *et al*. 2017). The balance between activation and inactivation results in a ‘sodium window current’ that determines cellular excitability.

Na_V_ channels consist of a pore‐forming α­subunit, which can form heterodimers or heterotrimers with auxiliary β­subunits that modify their gating properties (Calhoun & Isom, 2014; Kruger & Isom, 2016). Insulin‐secreting β‐cells are equipped with Na_V_ channels and express Na_V_1.3, Na_V_1.6 and Na_V_1.7 α‐subunits that are encoded by *Scn3a*, *Scn8a* and *Scn9a* genes, respectively. Moreover, they principally express *Scn1b*, which encodes the β_1_­subunit (Benner *et al*. 2014; Adriaenssens *et al*. 2016; DiGruccio *et al*. 2016).

In mouse and rat (but not in human) β‐cells, Na_V_ channels exhibit an unusual voltage dependence of inactivation (Hiriart & Matteson, 1988; Lou *et al*. 2003; Braun *et al*. 2008; Zhang *et al*. 2014). In most mouse β‐cells, inactivation proceeds at unphysiologically negative membrane potentials such that no Na^+^ voltage‐gated currents remain activatable at membrane potentials above −70 mV. In β‐cells, full Na_V_ reactivation requires membrane potentials as negative as ∼−120 mV. This is 40–50 mV more negative than the most repolarized membrane potential of the β­cell. As a result, most Na_V_ channels are ‘locked’ in the non‐conducting inactivated state (Plant, 1988; Gopel *et al*. 1999). However, more recently it was reported that inactivation in β‐cells is biphasic and consists of an additional small Na^+^ current component (10–15% of the total Na^+^ current) that persists at physiologically relevant membrane potentials in one‐third of the β‐cells (Vignali *et al*. 2006; Zhang *et al*. 2014).

It has been proposed that the two components of inactivation reflect different Na^+^ channel subtypes (Vignali *et al*. 2006; Zhang *et al*. 2014). Indeed, we have shown that whereas Na_V_1.7 gives rise to the component inactivating at hyperpolarized voltages, Na_V_1.3 accounts for the component inactivating over more physiological membrane potentials (Zhang *et al*. 2014). This would suggest that Na_V_1.7 channels in β‐cells inactivate at 40 mV more negative membrane potentials than in other cells and that β‐cells contain a factor modulating Na_V_1.7 channels in a subtype‐specific fashion.

Here we have compared the inactivation properties of different Na_V_ channel subtypes when expressed in insulin‐secreting cells and in HEK, CHO and glucagon‐secreting αTC1‐6 cells. To isolate the expressed current, we generated a tetrodotoxin (TTX)‐resistant form of the channels and blocked endogenous channels by inclusion of TTX in the bath medium. Our data confirm that Na_V_1.7 currents do indeed inactivate at more hyperpolarized membrane potentials than Na_V_1.3 and Na_V_1.6 in Ins1 cells but that the difference is small and insufficient to explain the biphasic inactivation observed in pancreatic β‐cells. Intriguingly, expression of a single Na_V_ subtype gives rise to currents that undergo biphasic inactivation over distinct and widely separated membrane potential ranges in individual Ins1 cells but never in the other cell types. We propose a model that accounts for the α­subunit‐independent biphasic inactivation of Na_V_ channels in β‐cells.

## Methods

### Ethical approval

With the exception of Fig. 13, all measurements were made in cell lines. Experiments in Fig. 13 were conducted in accordance with the UK Animals (Scientific Procedures) Act 1986 and University of Oxford ethical guidelines.

### Mice and islet isolation

The procedures for islet isolation, primary cell culture and generation of the *Scn3a* knockout mice were as described previously (Zhang *et al*. 2014).

### Plasmid constructs

The constructs coding for human brain *SCN9A* isoform, for human *SCN1B* and *SCN2B* expressed in tandem and for human *SCN3B* were kindly provided by Frank Reimann (University of Cambridge, UK) (Cox *et al*. 2006). The constructs for β­subunit expression also coded for green fluorescent protein (GFP), thus allowing tracing of transfected cells.

The constructs coding for human *SCN3A* (NM_006922), *SCN5A* (NM_198056) and *SCN8A* (NM_014191) bearing a Myc‐DDK‐tag at the C‐terminus were purchased from OriGene Technologies, Inc. (Rockville, MD, USA).

The α‐subunits of Na_V_1.3, Na_V_1.6 and Na_V_1.7 were rendered TTX‐resistant by replacing the amino acid tyrosine with serine at positions 384, 371 and 362, respectively (Cummins *et al*. 2001). Site‐directed mutagenesis was achieved using the Q5 Site‐Directed Mutagenesis Kit (New England Biolabs, Ipswich, MA, USA) with back‐to‐back primers according to the manufacturer's instructions. To generate Na_V_1.7–Na_V_1.3 hybrid constructs, N­terminal cytoplasmic domain, loop 1 (L1) and loop 2 (L2), loop 3 (L3) and the C‐terminal cytoplasmic domain of Na_V_1.7 (see Fig. 1) were replaced with the corresponding sequences of Na_V_1.3 using overlap PCR and Gibson assembly techniques. All mutations and the integrity of the open reading frames (ORFs) were verified by Sanger sequencing.

Figure 1Generation of Na_V_1.7 and Na_V_1.3 chimera constructsCLUSTAL format alignment of Na_V_1.7 (NP_002968) and Na_V_1.3 (NP_008853) amino acid sequences was performed using MAFFT software. Corresponding residue numbers are labelled on the left. Sequences of the generated chimeric α­subunits are shown at the bottom. Only partial alignments of the regions where changes were introduced are depicted. Na_V_1.3 sequences introduced into Na_V_1.7 backbone are highlighted in yellow. [Color figure can be viewed at http://wileyonlinelibrary.com]

**Figure:**
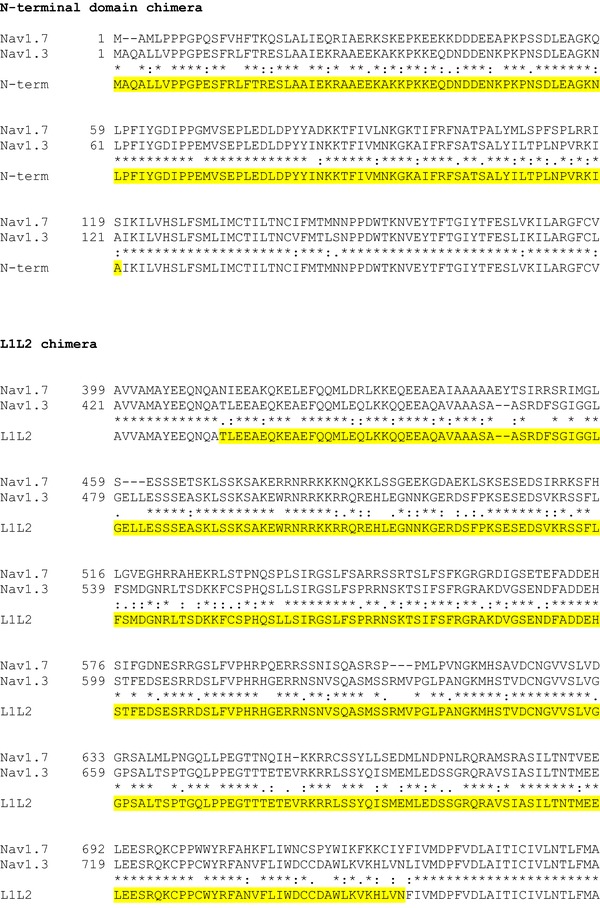

**Figure:**
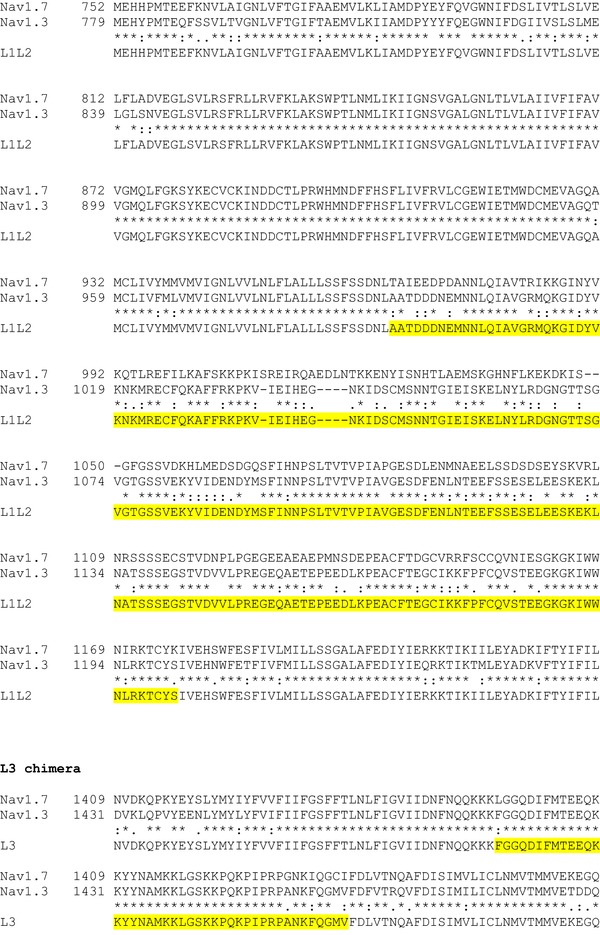

**Figure:**
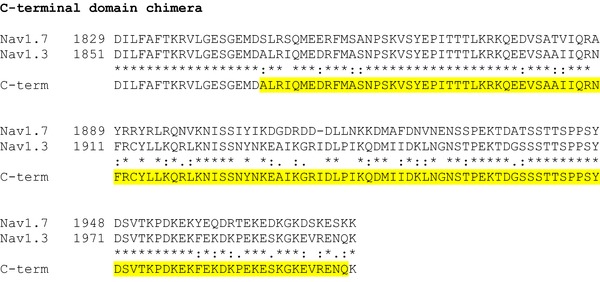

### Cell culture

The rat insulinoma cell line Ins1 832/13 (referred to as Ins1) was provided by J. Lang (Université de Bordeaux, France) and cultured in RPMI supplemented with 11 mm glucose (also 5 mm and 25 mm glucose in the indicated experiments), 10% fetal calf serum, 10 mm HEPES, 1 mm sodium pyruvate, 50 μm β‐mercaptoethanol and 100 U ml^−1^ penicillin and 100 μg ml^−1^ streptomycin. In specified experiments Ins1 cells were cultured for 48 h with 10 nm phorbol 12‐myristate 13‐acetate (PMA) (Sigma‐Aldrich, St Louis, MO, USA) or 100 μm diazoxide (Sigma‐Aldrich); or for 24 h with the insulin receptor antagonist S961 (Sigma‐Aldrich). The human embryonic kidney cell line (AD‐293; referred to as HEK cells from here on) was obtained from Agilent Technologies (Santa Clara, CA, USA), the mouse pancreatic α‐cell line (αTC1‐6) was obtained from ATCC (Manassas, VA, USA), the Chinese hamster ovary cell line (CHO) was obtained from European Collection of Authenticated Cell Cultures (Salisbury, UK). All cell lines were cultured according to the providers’ protocols.

Transfection of plasmids and small interfering RNA (siRNA) duplexes was performed using Lipofectamine® 2000 (Thermo Fisher Scientific, Waltham, MA, USA) according to the manufacturer's guidelines.

Cells plated on 35 mm dishes were co‐transfected with 1.5 μg of DNA encoding Na_V_1.3, Na_V_1.6 and Na_V_1.7 α­subunits and 50 ng of DNA encoding β­subunits (as specified) and assayed 24–48 h after transfection. GFP fluorescence was used to select for transfected cells, which subsequently were tested for channel expression by whole‐cell patch‐clamp recording techniques.

siRNA‐mediated knockdown experiments were performed in Ins1 cells. siRNA duplexes against rat *Scn3b* and scrambled negative control (OriGene Technologies, Inc.) were applied at a final concentration of 60 nm. For efficient knockdown, the cells were transfected on day 1 and day 3 and used for experiments on day 4. The efficiency of knockdown was assessed by qPCR.

### RNA isolation and quantitative RT‐PCR

RNA was isolated using a combination of TRI reagent and Ambion PureLink RNA Mini Kit (Thermo Fisher Scientific). On‐column DNase treatment was performed to eliminate genomic DNA contamination. cDNA was synthesized using the High Capacity RNA‐to‐cDNA Kit (Thermo Fisher Scientific). Real‐time qPCR was performed using SYBR Green detection and gene specific QuantiTect Primer Assays (Qiagen, Hileden, Germany). Relative expression was calculated using the Δ*C*_t_ method. Glyceraldehyde 3‐phosphate dehydrogenase (GAPDH) and peptidylprolyl isomerase A (PPIA) were used as reference genes.

### Electrophysiological recordings

Whole‐cell Na^+^ currents were recorded from untreated or transfected Ins1, HEK, αTC1‐6 and CHO cells using the standard whole‐cell configuration as previously described (Zhang *et al*. 2014). Voltage‐clamp experiments were performed using an EPC‐9 amplifier and Pulse (Version 8.80) software (HEKA Electronik, Lambrecht/Pfalz, Germany). A DMZ‐Zeitz‐Puller (Zeitz, Martinsreid, Germany) was used for fabrication of polished patch‐clamp electrodes (Harvard Apparatus, Cambridge, MA, USA) that had a resistance of 2–4.5 MΩ when filled with the electrode‐filling solution. Capacitive transients were compensated for using a computer‐controlled algorithm. The remaining capacitive transients as well as leak subtraction were removed using a –*P*/4 protocol. Series resistance compensation between 50 and 80% was used every 2 μs. The recorded currents were filtered by 2.9 kHz and digitized at >10 kHz. Recordings of cells with a seal resistance of less than 1GΩ, a voltage error greater than 5 mV and currents less than 50 pA (except in Fig. 7) were not used. Since Na^+^ current activation and inactivation undergo a time‐dependent shift towards more negative membrane potentials in the whole‐cell configuration (Fernandez *et al*. 1984), all measurements reported here were obtained within ∼1 min of achieving the whole‐cell configuration.

The standard extracellular medium for the electrophysiological measurements consisted of (mm): 118 NaCl, 20 tetraethylammonium‐Cl (TEA‐Cl), 5.6 KCl, 1.2 MgCl_2_, 5 HEPES, 5 d‐glucose and 2 CoCl_2_ (to block Ca^2+^ channels; also used at 0.2 and 10 mm final concentrations in the indicated experiments), adjusted to pH 7.4 using NaOH. TTX (Alomone Labs, Jerusalem, Israel) was used at a final concentration of 0.1 μg ml^−1^, to block endogenous Na^+^ currents. In the specified experiments BIM23056 (Tocris Bioscience, Bristol, UK) or PMA was present in the extracellular solution at a 100 nm and 10 nm final concentration, respectively. The pipette solution contained (mm): 120 CsCl, 1 MgCl_2_.6 H_2_O, 1 CaCl_2_, 10 EGTA, 10 HEPES) and 3 Mg‐ATP, adjusted to pH 7.15 with CsOH. In the indicated experiments, phosphatidylinositol 4,5‐bisphosphate (PIP_2_) diC8 (Echelon Bioscience Inc, Salt Lake City, UT, USA) or neomycin (Sigma‐Aldrich) was present in the pipette solution at 50 μm final concentration.

The effect of acute changes in the extracellular glucose concentration were measured in the perforated‐patch configuration, as previously described (De Marinis *et al*. 2010). Perforation was achieved using amphotericin B (Sigma‐Aldrich), present in the intracellular solution at 0.4 mg ml^−1^ final concentration. The intracellular pipette solution was composed of (mm): 76 Cs_2_SO_4_, 10 NaCL, 10 KCl, 1 MgCl_2_ and 5 Hepes, adjusted to pH 7.15 with CsOH. The extracellular solution used in the whole‐cell configuration was also used for perforated patch clamp measurements at a final glucose concentration of 1 or 20 mm.

For cell‐attached experiments, the pipette solution had an extracellular composition of (mm): 118 NaCl, 20 tetraethylammonium‐Cl, 5.6 KCl, 1.2 MgCl_2_, 5 HEPES, 2 CoCl_2_ and 5 d‐glucose, adjusted to pH 7.4 with NaOH. During seal formation, the cells were immersed in standard extracellular medium containing (mm): 137 NaCl, 5.6 KCl, 10 Hepes (pH 7.4 using NaOH), 1.1 MgCl_2_ and 2.6 CaCl_2_. Once the seal was formed, the high K^+^ extracellular solution was perfused, consisting of (mm): 125 KCl, 1 MgCl_2_, 1 CaCl_2_, 10 EGTA, 10 Hepes and 5 d‐glucose, adjusted to pH 7.4 with KOH to depolarize the cell to ∼0 mV (Nernst potential) to allow accurate control of the membrane potential. After recordings were made in the cell‐attached configuration, an extracellular solution with an intracellular ion composition was perfused, ready for patch excision into an inside‐out configuration. The extracellular medium used for the inside‐out patch experiments was identical to the high‐K^+^ solution specified above except that pH was adjusted to 7.2 and 3 mm Mg­ATP was added. All electrophysiological experiments were performed at 34°C.

For ‘patch cramming’ experiments, Ins1 and HEK cells were cultured in droplets of their respective culture media in the same 35 mm dish. The same solutions that were used for cell‐attached and inside‐out configurations were then applied. However, upon excision of the membrane, the electrode was ‘crammed’ into a neighbouring HEK cell followed by subsequent measurements of voltage‐dependent activation and inactivation (Kramer, 1990).

A standard two‐pulse protocol was used to assess inactivation. The pulse protocol consisted of a 50 ms conditioning pulse of potentials varying from −150 to 0 mV (in increments of 5 mV) followed by a 5 ms test pulse to 0 mV. The peak inward current (*I*) produced after each depolarization to 0 mV was measured and normalized to the maximum Na^+^ current amplitude (*I*_max_). The data are presented as *h*_∞_ (= *I*/*I*_max_), which was plotted against the conditioning voltage. For each cell, the data points were approximated to a single or a double Boltzmann function to determine the half‐maximal inactivation (*V*_h_) and the slope factor (*k*). The most appropriate fit to the data (single or double Boltzmann) was determined using the Akaike information criterion using Origin software (OriginLab Corp., Northampton, MA, USA). Inactivation curves were fitted to a single Boltzmann function when the fit to a double Boltzmann function resulted in either two *V*_h_ values with a difference of less than 15 mV or a *V*_h_ composed of less than 15% of the total current.

Steady‐state activation was assessed by applying a depolarizing pulse, between −70 and +60 mV (in increments of 10 mV) for 20 ms, from a holding potential of −150 mV. The peak inward current (*I*) produced after depolarization to each voltage step was measured and used to calculate the Na^+^ conductance (*G*) by the relation *G = I*/(*V* − *V*_r_) where *V* is the membrane potential and *V*_r_ is the estimated reversal potential, calculated using the Nernst equation from the extra‐ and intracellular Na^+^ concentrations in the media (Gonoi & Hille, 1987). The sigmoidal curve produced was fitted to a Boltzmann function, which was used to determine the half‐maximal activation (*V*_h_) and the slope factor (*k*).

### Data analysis

All data are given as mean values ± SEM of the indicated number of experiments (*n*). Statistical significances were calculated using Student's *t* test or ANOVA (for multiple comparisons, as appropriate).

## Results

### Characterization of TTX‐resistant Na^+^ channels

**Figure 2:**
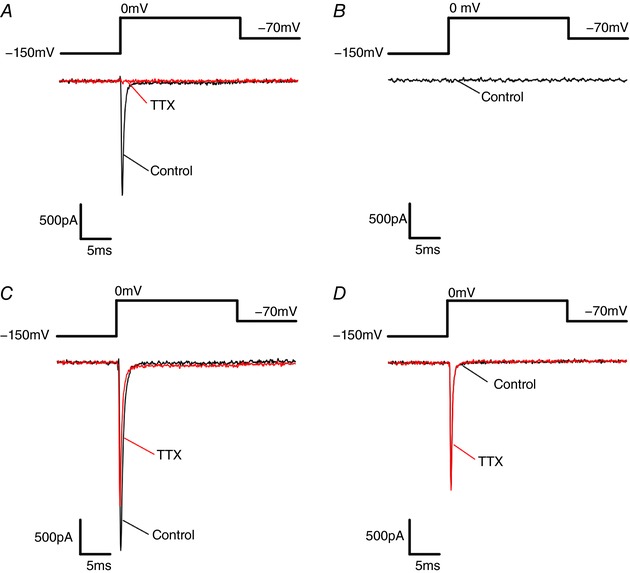
Endogenous Na_V_ and mutant TTX‐resistant Na_V_1.7 currents in Ins1 and HEK cells *A*, endogenous Na_V_ current from Ins1 cells in response to a step depolarization from −150 to 0 mV in the absence (black; control) and presence of TTX (red; TTX treated). *B*, no endogenous Na_V_ currents were evoked in HEK cells by a step depolarization from −150 to 0 mV. *C*, Na_V_ currents evoked in response to a step depolarization from −150 to 0 mV in Ins1 cells co‐transfected with mutant TTX‐resistant Na_V_1.7 and β_1_‐ and β_2_‐subunits in the absence (black; control) and presence of TTX (red; TTX treated). The reduction of the peak current induced by TTX reflects block of endogenous Na_V_ current. *D*, same as in *C* but expressed in HEK cells. [Color figure can be viewed at http://wileyonlinelibrary.com]

To further explore the role of the different Na^+^ channel α­subunits and their contribution to voltage dependence of inactivation, it was important to isolate the current from individual Na_V_ channel α­subunits. As there are currently no reliable α­subunit‐specific Na^+^ blockers, we generated TTX‐resistant α­subunits by site­directed mutagenesis (see Methods) and expressed them in clonal β‐cells and HEK cells. Figure 2 *A* and *B* shows Na^+^ currents recorded from non‐transfected Ins1 and HEK cells during a voltage‐clamp depolarization to 0 mV. All untransfected Ins1 cells contained TTX‐sensitive voltage‐gated Na^+^ currents (Na_V_ currents; *n* = 21). By contrast, none of the HEK cells contained any Na_V_ currents. After transfection of the cells with mutant Na_V_1.7, large TTX‐resistant Na_V_ currents were observed in both Ins1 and HEK cells (Fig. 2 *C* and *D*). Similar data were obtained when cells were transfected with TTX‐resistant Na_V_1.3 and Na_V_1.6 or wild‐type Na_V_1.5 – a naturally TTX‐resistant channel (data not shown). It was ascertained in HEK cells that making the Na_V_1.3, Na_V_1.6 and Na_V_1.7 channels TTX resistant did not affect voltage dependence of inactivation (not shown). From here on the channels made resistant to TTX will be referred to simply as Na_V_1.7, Na_V_1.3 and Na_V_1.6.

### Inactivation of Na_V_1.3 and Na_V_1.7 expressed in Ins1 cells

**Figure 3:**
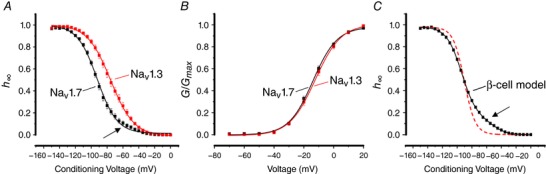
Voltage dependence of inactivation and activation of Na_V_1.7 and Na_V_1.3 in Ins1 cells *A*, voltage dependence of inactivation (*h*_∞_) of Na_V_1.7 (black; *n = *47) and Na_V_1.3 (red; *n = *34) when the α‐subunit is co­expressed with β_1_‐ and β_2_‐subunits in Ins1 cells. The curves represent a single Boltzmann fit to the data. Note slight deviation from inactivation at membrane potentials between −70 and −40 mV in Ins1 cells expressing Na_V_1.7 (arrow). *B*, voltage dependence of Na_V_1.7 (black; *n = *39) and Na_V_1.3 (red; *n = *31) current activation (*G*/*G*_max_) in Ins1 cells. The curves represent a single Boltzmann fit to the data. *C*, the black curve represents a model of β‐cell Na_V_ current inactivation behaviour composed of 15% Na_V_1.3 and 85% Na_V_1.7, fitted to a double Boltzmann function. Superimposed is a single Boltzmann fit to the model (dashed red). The β‐cell model exhibits a clear shoulder at conditioning membrane potentials of −70 to −40 mV (arrow). See also Table 1. [Color figure can be viewed at http://wileyonlinelibrary.com]

We expressed Na_V_1.3 or Na_V_1.7 in Ins1 cells and determined their voltage dependence of activation and inactivation, which were described by fitting *single* Boltzmann functions to the data points (Table 1). The two types of Na_V_ channel α­subunit exhibited rather different inactivation behaviours, and *V*_h_ averaged −76 ± 2 (*n* = 34) and −92 ± 2 mV (*n* = 47) for Na_V_1.3 and Na_V_1.7, respectively (Fig. 3 *A*). However, it is noticeable that the inactivation of Na_V_1.7 in Ins1 cells shows some slight deviation from a single Boltzmann function (arrow). Although the inactivation of Na_V_1.3 appears to be monophasic, inactivation in individual cells was clearly best described using a double Boltzmann fit to the data. This aspect will be addressed further below (Fig. 11).

**Table 1:** *V*_h_ and *k* values of Na_v_ channel inactivation in Ins1, HEK, CHO and αTC1‐6 cells

	Na_V_1.7		Na_V_1.3		Na_V_1.6		Na_V_1.5	
Cell type	*V*_h_ (mV)	*k*	*V*_h_ (mV)	*k*	*V*_h_ (mV)	*k*	*V*_h_ (mV)	*k*
Ins1	−92 ± 2 (47)	10 ± 0.4 (47)	−76 ± 2 (34)	12 ± 1 (34)	−73 ± 2 (35)	10 ± 0.3 (35)	−99 ± 2 (9)	8 ± 0.3 (9)
HEK	−66 ± 2 (9)	8 ± 0.5 (9)	−48 ± 1 (7)	10 ± 2 (7)	−53 ± 1 (10)	7 ± 0.4 (10)	−77 ± 1 (10)	6 ± 0.2 (10)
CHO	−61 ± 3 (10)	9 ± 1 (10)	−50 ± 2 (13)	7 ± 0.4 (13)	n/a	n/a	n/a	n/a
αTC1‐6	−68 ± 3 (10)	7 ± 1 (10)	−59 ± 4 (9)	6 ± 0.5 (9)	n/a	n/a	n/a	n/a

The *V*_h_ and *k* values of Na_V_ α‐subunits co‐expressed with β_1_‐ and β_2_‐subunits. Data were fitted to a single Boltzmann function. Values represent means ± SEM of indicated number of cells (*n*).

By contrast, activation of the two α‐subunits was essentially superimposable and the half‐maximal activation is −12 ± 1 (*n* = 39) and −13 ± 1 mV (*n* = 31) for Na_V_1.3 and Na_V_1.7, respectively (Fig. 3 *B*).

We have previously proposed that biphasic inactivation of the Na^+^ current in primary β‐cells reflects the expression of Na_V_1.3 and Na_V_1.7 α‐subunits and that the two subunits underlie the positive and negative inactivation components, respectively. We modelled Na^+^ current inactivation behaviour in a cell containing 15% Na_V_1.3 and 85% Na_V_1.7 (the observed relative contribution of the positive and negative inactivation components in primary β‐cells (Zhang *et al*. 2014). As shown in Fig. 3 *C*, the resulting inactivation curve thus obtained exhibits a clear shoulder at conditioning membrane potentials of −70 to −40 mV (arrow). We conclude that expression of different α‐subunits may contribute to the biphasic inactivation observed in primary β‐cells. However, as will be explained below, this is not the only contributing factor.

### Comparison of Na_V_1.3, Na_V_1.5, Na_V_1.6 and Na_V_1.7 inactivation in Ins1 and HEK cells

**Figure 4:**
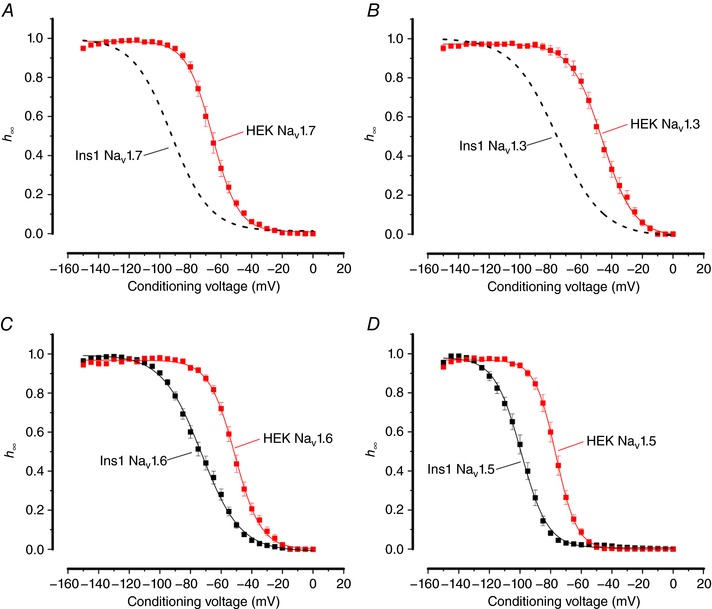
Voltage dependence of inactivation of Na_V_1.7, Na_V_1.3, Na_V_1.6 and Na_V_1.5 currents in Ins1 cells compared to HEK cells *A*, voltage dependence of Na_V_1.7 current inactivation (*h*_∞_) when the α‐subunit is co‐expressed with β_1_‐ and β_2_‐subunits in Ins1 (same data as in Fig. 3 *A*: dashed curve) and HEK cells (red; *n = *9). The curve represents a single Boltzmann fit to the data. *B*, as in *A* but for Na_V_1.3 (dashed curve same data as in Fig. 3 *A*; red, *n = *7). *C*, as in *A* but for Na_V_1.6 (black, *n = *35; red, *n = *10). *D*, as in *A* but for Na_V_1.5 (black, *n = *9; red, *n = *10). See also Table 1. [Color figure can be viewed at http://wileyonlinelibrary.com]

The inactivation of Na_V_1.7 in Ins1 cells (*V*_h_ = −93 mV) is more negative than what has been reported previously in HEK cells and in neurones (Herzog *et al*. 2003; Eberhardt *et al*. 2014). We next systematically compared inactivation of Na_V_1.3, Na_V_1.5, Na_V_1.6 and Na_V_1.7 co‐expressed with β_1_ and β_2_ in Ins1 and HEK cells (Fig. 4 *A*–*D*). We found that for all α­subunits, inactivation (expressed as *V*_h_) occurred at membrane potentials 20–30 mV more negative in Ins1 cells than in HEK cells or CHO cells and that inactivation of Na_V_1.7 occurred at 10–20 mV more negative membrane potentials than Na_V_1.3 and Na_V_1.6 regardless of the cell type (Table 1).

**Figure 5:**
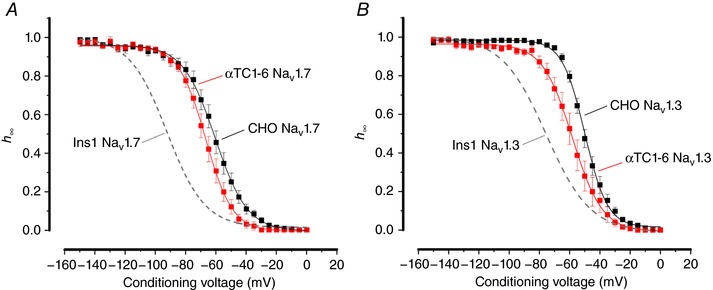
Voltage dependence of inactivation of Na_V_1.7 and Na_V_1.3 currents in αTC1‐6 and CHO cells *A*, voltage dependence of inactivation of Na_V_1.7 currents when the α‐subunit is co­expressed with β_1_‐ and β_2_‐subunits in αTC1‐6 (red; *n = *10) and CHO (black; *n = *10) cells. The curve represents a single Boltzmann fit to the data and the grey dashed line represents the inactivation of Na_V_1.7 expressed in Ins1 cells (shown in Fig. 3). *B*, same as in *A*, but experiments were conducted with Na_V_1.3 in αTC1­6 (red; *n = *9) and CHO cells (black; *n = *13). See also Table 1. [Color figure can be viewed at http://wileyonlinelibrary.com]

Inactivation properties of both Na_V_1.3 and Na_V_1.7 expressed in CHO cells were similar to that observed in HEK cells (Fig. 5 *A* and *B* and Table 1). In the glucagon­secreting cell line αTC1‐6, inactivation of both Na_V_1.3 and Na_V_1.7 was more similar to that found in HEK and CHO cells than in the insulin‐secreting cells (Fig. 5 *A* and *B* and Table 1).

### Impact of β‐subunits on Na_V_ inactivation

**Figure 6:**
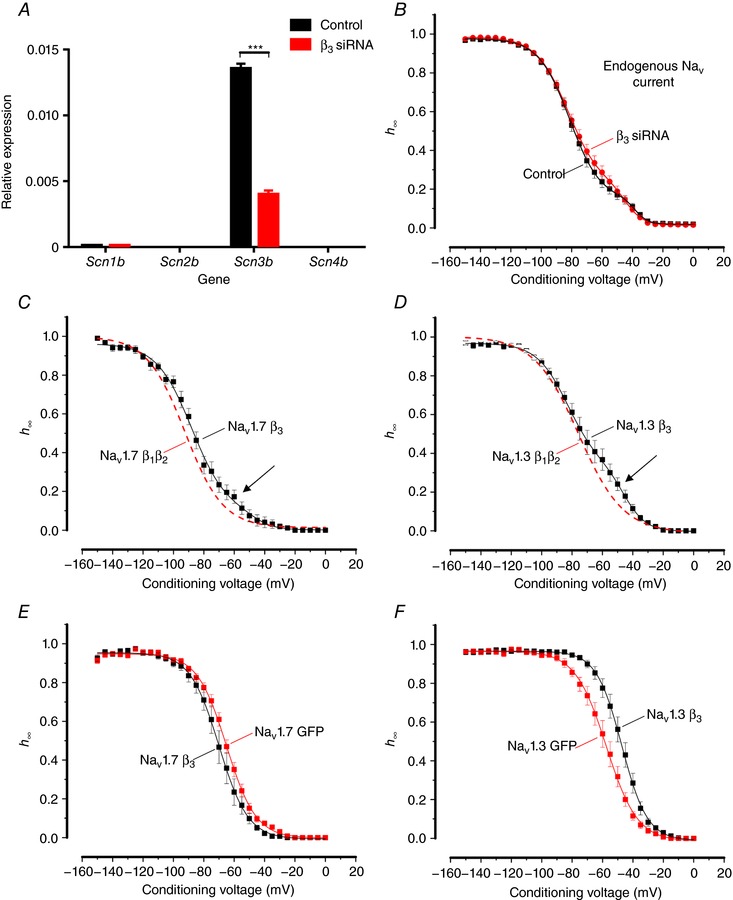
Effect of β‐subunits on Na_V_ current inactivation in Ins1 and HEK cells *A*, relative mRNA expression of *Scn1b*, *Scn2b*, *Scn3b* and *Scn4b* in control Ins1 cells transfected with a scramble siRNA or β_3_ siRNA (*n = *3 preparations). *B*, voltage dependence of inactivation (*h*_∞_) of endogenous Na_V_ currents from control (black; *n = *22, from 3 preparations) and β_3_ siRNA treated Ins1 cells (red; *n = *26 from 3 preparations). The curves represent a double Boltzmann fit to the data. *C*, voltage dependence of Na_V_1.7 current inactivation when the α‐subunit is co‐expressed with β_3_‐subunit (black; *n = *9) in Ins1 cells. Dashed red curve represents voltage dependence of Na_V_1.7 current inactivation when the α‐subunit is co‐expressed with β_1_‐ and β_2_‐subunits in Ins1 cells (data shown in Fig. 3). The curves represent a double Boltzmann fit to the data. Arrow indicates a component of inactivation occurring at positive membrane potentials. *D*, as in *C* but for Na_V_1.3 (black; *n = *11). *E*, voltage dependence of Na_V_1.7 current inactivation when the α­subunit is co­expressed with β_3_‐subunit (black; *n = *9) or GFP alone (red; *n = *12) in HEK cells. The curves represent a single Boltzmann fit to the data. *F*, as in *E*, but for Na_V_1.3 (black, *n = *12; red; *n = *10). *V*_h_ and *k* value for the respective conditions are given in Table 2. [Color figure can be viewed at http://wileyonlinelibrary.com]

The Na_V_ channel β‐subunits modulate inactivation of Na_V_ α‐subunits (Cummins *et al*. 2001; Calhoun & Isom, 2014). Moreover, as the β‐subunits are prone to cell‐specific post‐translational modulation, their effect on Na_V_ inactivation may be cell specific, i.e. the same β‐subunit can have different effects on Na_V_ inactivation in different cell types (Isom *et al*. 1992, Moran *et al*. 2000; Meadows & Isom, 2005). To test whether modulation of β‐subunits is the mechanism by which Na_V_ currents in Ins1 cells inactivate at more hyperpolarized potentials compared to other cells types, siRNA knockdown was used to ablate β‐subunit expression in Ins1 cells. The only β‐subunit expressed endogenously in Ins1 cells is the β_3_­subunit (Fig. 6 *A*). At the mRNA level, 71% knockdown of *Scn3b* was achieved with no compensatory increase in other β‐subunits (i.e. *Scn1b*, *2b* or *4b*). Down‐regulation of *Scn3b* only marginally affected the inactivation of the endogenous Na^+^ current in Ins1 cells (Fig. 6 *B*, Table 2). The inactivation of the endogenous Na_V_ current was clearly biphasic but this was not affected by down‐regulating the β_3_­subunit.

**Table 2:** Effect of β­subunits on Na_V_ current inactivation in Ins1 and HEK cells

		Monophasic		Biphasic			
Cell type	Na_V_ current	*V*_h_ (mV)	*k*	*V*_h1_ (mV)	*k*_1_	*V*_h2_ (mV)	*k*_2_
Ins1	Endogenous control	−81 ± 2 (11)	11 ± 1 (11)	−81 ± 1 (11)	9 ± 1 (11)	−43 ± 3 (11)	6 ± 2 (11)
Ins1	Endogenous β_3_ siRNA	−82 ± 3 (9)	10 ± 1 (9)	−83 ± 2 (17)	10 ± 1 (17)	−47 ± 2 (17)	6 ± 1 (17)
Ins1	Na_V_1.7 + β_3_	−89 ± 2 (8)	11 ± 1 (8)	−86 (1)	6 ± 1 (1)	−68 (1)	14 (1)
Ins1	Na_V_1.3 + β_3_	−72 ± 10 (3)	9 ± 2 (3)	−84 ± 2 (8)	11 ± 1 (8)	−46 ± 2 (8)	5 ± 1 (8)
HEK	Na_V_1.7 + β_3_	−70 ± 3 (9)	8 ± 1 (9)				
HEK	Na_V_1.7 + GFP	−66 ± 2 (12)	9 ± 1 (12)				
HEK	Na_V_1.3 + β_3_	−48 ± 2 (12)	7 ± 1 (12)				
HEK	Na_V_1.3 + GFP	−59 ± 3 (10)	8 ± 1 (10)				

Values represent means ± SEM of indicated number of cells (*n*). See Fig. 6. *V*_h_ represents membrane potential at which inactivation is half‐maximal in cells with monophasic inactivation and *k* represents the slope factor. In cells with biphasic inactivation, *V*_h1_ and *V*_h2_ represent the membrane potential at which inactivation is half‐maximal for the current components inactivating at negative and positive membrane potentials, respectively. *k*_1_ and *k*_2_ represent the respective slope factors for the currents inactivating with *V*_h1_ and *V*_h2_.

We also compared the inactivation of the expressed human Na_V_1.3 and Na_V_1.7 when co‐expressed with β_3_ (rather than β_1_ and β_2_, as in previous experiments) in Ins1 cells (Fig. 6 *C* and *D*). In both cases, overexpression of the β_3_‐subunit made biphasic inactivation more apparent (arrows).

Finally, we compared Na_V_1.3 and Na_V_1.7 inactivation when co‐expressed with or without β_3_ in HEK cells. Whereas the inactivation of Na_V_1.7 was shifted by 4 mV towards more negative voltages in the presence of β_3_, inactivation of Na_V_1.3 was shifted 11 mV towards more depolarized membrane potentials (Fig. 6 *E* and *F*). It was ascertained that HEK cells do not express any endogenous β­subunits (not shown).

Collectively, these data suggest that the widely different inactivation behaviours of Na_V_1.3 and Na_V_1.7 when expressed in Ins1 and HEK cells cannot be attributed to any of the differential modulation of β­subunits in a cell‐specific manner, expression of a different endogenous β‐subunit complement in Ins1 cells or the α­subunits forming heterodimers with different β‐subunits (in which case overexpression of β_3_ should have made biphasic inactivation less apparent). Rather the data suggest that the hyperpolarized inactivation observed in β‐cells is due to a direct effect on the Na_V_ channel α‐subunit. Moreover, we conclude that the biphasic inactivation of the endogenous Na^+^ current in Ins1 cells need not reflect the presence of multiple α‐subunits as biphasic inactivation was also observed in the experiments involving expression of Na_V_1.3 and Na_V_1.7 alone.

### Negative Na_V_ inactivation in Ins1 cells is not due to cytosolic diffusible factor

**Figure 7:**
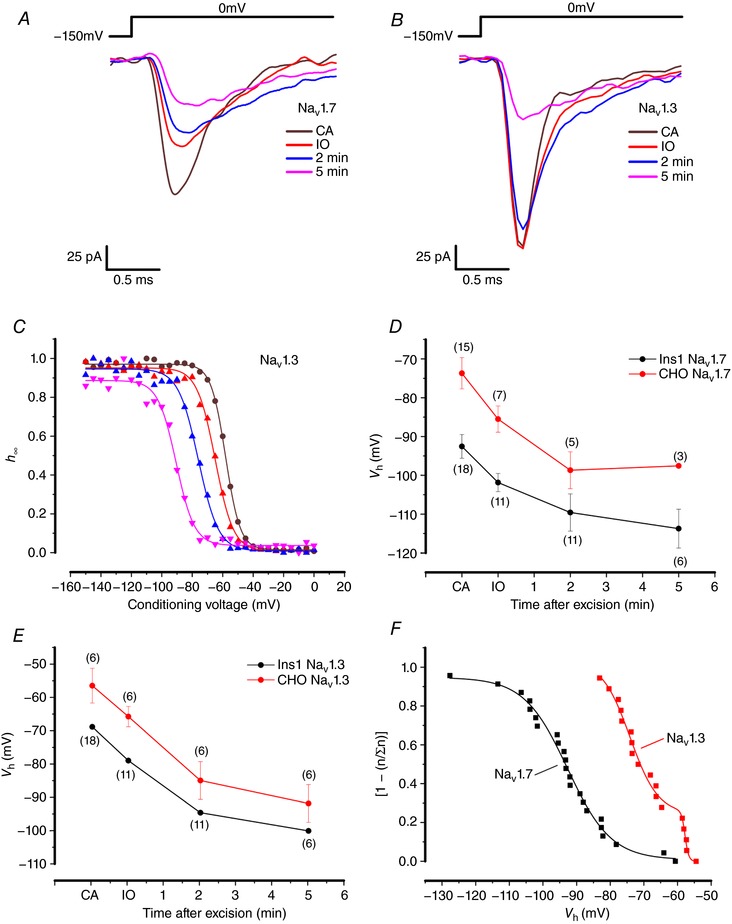
Current inactivation of Na_V_1.7 and Na_V_1.3 in cell‐attached and inside‐out configuration *A*, Na_V_1.7 currents when the α‐subunit is co‐expressed with β_1_ and β_2_ in Ins1 cells in response to a step depolarization from −150 to 0 mV in cell‐attached (CA), inside‐out (IO) and 2 and 5 min after excision. *B*, same as in *A*, but with Na_V_1.3 currents in Ins1 cells. *C*, voltage dependence of inactivation of Na_V_1.3 currents shown in *B* (same colour code). *D*, summarized voltage dependence of inactivation of Na_V_1.7 currents in Ins1 (black) and CHO cells (red) of indicated number of patches/cells (*n*). *E*, same as in *D* but for Na_V_1.3. *F*, cumulative distribution of Na_V_1.3 (red) and Na_V_1.7 (black) *V*_h_ recorded in cell‐attached patches on Ins1 cells. Data have been normalized (*n*/∑*n*) to the total number of patches (*n* = 18 patches for Na_V_1.3 and *n* = 23 patches for Na_V_1.7). For display and to facilitate comparison with inactivation curves, data are shown as (1 − [*n*/∑*n*]). The cumulative distributions have been fitted to a double Boltzmann function for Na_V_1.3 and a single Boltzmann function for Na_V_1.7. [Color figure can be viewed at http://wileyonlinelibrary.com]

We hypothesized that the inactivation of Na_V_1.3 and Na_V_1.7 is shifted towards more negative membrane potentials in Ins1 cells (and mouse β‐cells) compared to other cells (dorsal root ganglion neurones, HEK, CHO, αTC1‐6) because of the presence of a cytoplasmic factor in β‐cells that is not present in other cells. To explore this possibility, Na_V_1.3 and Na_V_1.7 were expressed in Ins1 cells and CHO cells and inactivation was measured in cell‐attached and subsequently in inside‐out patch configuration (to promote rapid ‘wash‐out’ of any attached modulators). These experiments required channel expression high enough to allow measurements of macroscopic (‘whole‐cell’) currents in cell‐attached patches (Fig. 7 *A* and *B*).

Figure 7 *C* shows inactivation curves for Na_V_1.3 currents recorded in cell‐attached patches and at various times after excision and formation of inside‐out patches in Ins1 cells. In the cell‐attached configuration, *V*_h_ for Na_V_1.7 was −92 ± 3 mV (*n* = 23) and −74 ± 4 mV (*n* = 15) in Ins1 and CHO cells, respectively. The corresponding values for Na_V_1.3 were −69 ± 2 (*n* = 18) and −56 ± 5 mV (*n* = 6) in Ins1 and CHO cells, respectively. We acknowledge that these values are not identical to those measured in the whole­cell configuration. In the cell‐attached experiments, the membrane potential of the cell was assumed to be 0 mV when the cells were immersed in the high‐[K^+^]_o_ medium, but it is possible that a slight voltage difference remains. This idea is supported by the observation that following excision of the patches, there was an immediate ∼10 mV shift in inactivation in the hyperpolarizing direction for both Na_V_1.3 and Na_V_1.7 expressed in either cell type.

In the cell‐attached patches, inactivation of Na_V_1.3 was invariably monophasic. However, the distribution at which Na_V_1.3 inactivated in the cell‐attached configuration appears to have two distinct components. Figure 7 *F* shows the normalized cumulative distribution of *V*_h_ measured in 18 membrane patches (each represented by a single point) in Ins1 cells expressing Na_V_1.3. The continuous curve represents a double Boltzmann fit to data points with *V*_h_ values of −74 mV and −58 mV. Five of the 18 patches (28%) had *V*_h_ values more positive than −60 mV. For Na_V_1.7 (black squares and line), the cumulative distribution for 23 patches was essentially monophasic with a *V*_h_ of −93 mV with only two patches (9%) showing a *V*_h_ at membrane potentials more positive than −65 mV.

If there is a diffusible modulator of Na_V_ inactivation present in β‐cells that shifts inactivation, then patch excision would result in a positive shift of inactivation (i.e. towards that observed in CHO cells). As already remarked, patch excision resulted in an immediate ∼10 mV shift of inactivation towards more hyperpolarized membrane potentials for both Na_V_1.3 and Na_V_1.7 and for both Ins1 and CHO cells. Following patch excision there was then a time‐dependent additional and parallel negative shift of *V*_h_ in both cell types until *V*_h_ eventually settled at −120 mV in Ins1 cells and −100 mV in CHO cells (Fig. 7 *D*). Similar time‐ and cell‐dependent changes were observed for Na_V_1.3 channels (Fig. 7 *E*). If diffusible factors modulating Na_V_ current inactivation were present in Ins1, then the curves would have been expected to converge. In addition, there was a time‐dependent decrease in current amplitude. We attribute this to rundown of channel activity, a process observed for many types of channels (Becq, 1996). Importantly, the shift in inactivation was nearly maximal 2 min after patch excision when effects on current kinetics were moderate (compare red and blue traces in Fig. 7 *A* and *B*).

Activation of the Na_V_1.3 and Na_V_1.7 channels underwent similar time‐dependent changes in the hyperpolarizing direct following patch excision (not shown).

**Figure 8:**
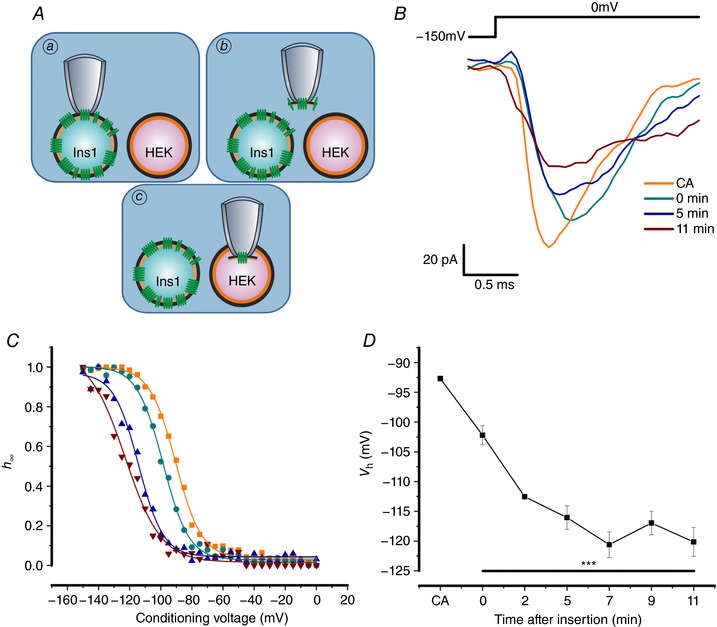
Na_V_1.7 expressed in Ins1 cells crammed into HEK cells *A*, schematic representation of patch cramming procedure. Na_V_1.7 channels were expressed in Ins1 cells and a cell‐attached patch was formed (*a*); the patch of membrane was excised (*b*) and immediately crammed into HEK cells (*c*). *B*, Na_V_1.7 currents when the α‐subunit is co‐expressed with β_1_‐ and β_2_­subunits in response to a step depolarization from −150 to 0 mV in cell‐attached (CA) configuration in Ins1 cells, immediately after excision and cramming into HEK cells (0 min) and 5 and 11 min after insertion of the membrane into HEK cells. *C*, voltage dependence of inactivation of Na_V_1.7 currents shown in *B* with same colour coding. *D*, summarized voltage dependence of inactivation of Na_V_1.7 currents in cell‐attached configuration and after cramming into HEK cells (*n = *4). ^***^ *P* < 0.001 for each time point compared to CA, using a one‐way ANOVA. [Color figure can be viewed at http://wileyonlinelibrary.com]

We also tested the alternative possibility that inactivation is more negative in Ins1 cells because something present in all other cells is missing in these cells. This was tested by an approach similar to that in Fig. 7 but instead of leaving the patch in the bath medium after excision, the patch was ‘crammed’ (Kramer, 1990) into a neighbouring HEK cell (Fig. 8 *A*). However, as shown in Fig. 8 *B* and *C*, the time‐dependent shift of *V*_h_ towards more negative membrane potentials persisted and was as pronounced after cramming the electrode into a HEK cell as when the patch was left in the bath medium; inactivation still underwent a 25 mV shift towards more negative membrane potentials 5–10 min after patch excision and cramming into the neighbouring HEK cell (Fig. 8 *D*).

### Cytoplasmic domains of Na_V_1.3 and Na_V_1.7 do not confer different inactivation behaviours

The data of Figs 7 and 8, suggest that the differences in inactivation of Na_V_ channels between Ins1 and HEK and CHO cells is unlikely to be attributable to freely diffusible factors present in the cytosol of either cell type. However, we acknowledge that the change in inactivation may result from firmer interaction between the Na_V_ α­subunits and another factor (protein) at the time they are inserted into the plasmalemma.

**Figure 9:**
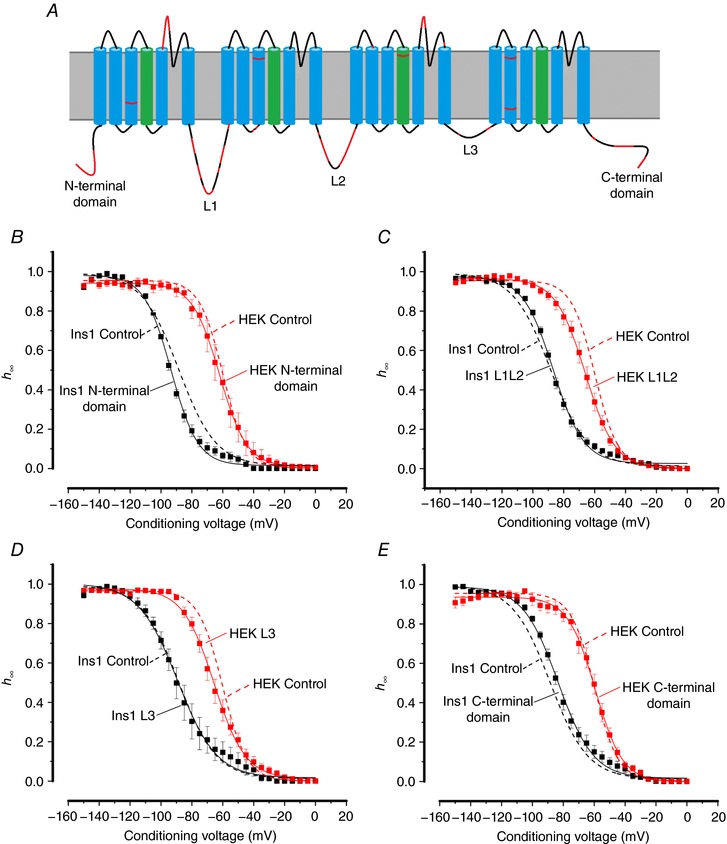
Na_V_1.7–Na_V_1.3 chimeras expressed in Ins1 and HEK cells *A*, schematic representation of the structure of the Na_V_1.7 channel. Highlighted in red are the areas of divergence in the amino acid sequence to Na_V_1.3. Sequence alignment was performed using the Clustal Omega program (http://www.uniprot.org/align/), using the NCBI reference sequences NP_002968.1 and NP_008853.3 for human Na_V_1.7 and Na_V_1.3, respectively. *B*, voltage dependence of inactivation of N‐terminal chimera currents when the α‐subunit is co‐expressed with β_1_‐ and β_2_‐subunits in Ins1 (black; *n = *9) and HEK cells (red; *n = *6); and control TTX‐resistant Na_V_1.7 currents when the α‐subunit is co‐expressed with β_1_‐ and β_2_­subunits in Ins1 (dashed black line; *n = *37) and HEK cells (dashed red line; *n = *6). *C*, as in *B* but for L1L2 chimera currents (black, *n = *15; red, *n = *9) with the same controls as in *B. D*, as in *B* but for L3 chimera currents (black, *n = *16; red, *n = *11) with the same controls as in *B*. *E*, as in *B* but for C‐terminal chimera currents (black, *n = *7; red, *n = *8) with the same controls as in *B. V*_h_ and *k* values are given in Table 3. [Color figure can be viewed at http://wileyonlinelibrary.com]

The schematic diagram in Fig. 9 *A* shows the topology of the Na_V_1.7 channels and highlights the areas of divergence in the amino acid sequence between Na_V_1.3 and Na_V_1.7. As indicated, the greatest differences are found in the N‐terminus, the C‐terminus and the cytoplasmic loops L1 and L2. We hypothesized that the sequence variations contribute both to differences in inactivation between (i) Na_V_1.3 and Na_V_1.7 and (ii) Ins1 and HEK cells. We acknowledge that this assumes that the differences in the intracellular/cytoplasmic milieu are transduced via the cytoplasmic domains of the channels. It follows from these premises that substituting the cytoplasmic domains of Na_V_1.7 for Na_V_1.3 will reduce the differences in *V*_h_ between the two α­subunits (i.e. change *V*_h_ from −92 mV to −76 mV in Ins1 cells; Table 1).

We generated chimeric channels based on the Na_V_1.7 backbone by replacing the N‐terminal domain, L1 and L2, the C‐terminal domain or L3 sequences with the corresponding sequences of Na_V_1.3 and analysed their properties in Ins1 (black) and HEK cells (red) (Fig. 9 *B*–*E*: see also Table 3). For comparison, the inactivation curves for wild‐type Na_V_1.7 in Ins1 and HEK cells are also shown (dashed black and red lines). None of the substitutions significantly affected Na_V_1.7 inactivation. Taken together with the data of Figs 7 and 8, it seems unlikely that the negative shift is caused by an intracellular factor interacting with the cytoplasmic domains of the Na_V_1.7 channels.

**Table 3:** Na_V_1.7–Na_V_1.3 chimeras expressed in Ins1 and HEK cells

		Monophasic		Biphasic			
Cell type	Na_V_ current of Na_V_1.7–1.3 chimeras	*V*_h_ (mV)	*k*	*V*_h1_ (mV)	*k*_1_	*V*_h2_ (mV)	*k*_2_
Ins1	Na_V_1.7 Control	−90 ± 2 (28)	9 ± 1 (28)	−94 ± 4 (9)	9 ± 1 (9)	−63 ± 5 (9)	8 ± 1 (9)
Ins1	N­terminal domain	−95 ± 1 (7)	7 ± 1 (7)	−98 ± 1 (2)	6 ± 1 (2)	−67 ± 1 (2)	12 ± 2 (2)
Ins1	L1L2	−88 ± 1 (12)	9 ± 1 (12)	−86 ± 1 (3)	7 ± 1 (3)	−49 ± 5 (3)	13 ± 7 (3)
Ins1	L3	−96 ± 3 (12)	8 ± 1 (12)	−98 ± 3 (4)	9 ± 2 (4)	−50 ± 5 (4)	9 ± 2 (4)
Ins1	C‐terminal domain	−86 ± 1 (5)	9 ± 1 (5)	−84 ± 1 (2)	10 ± 1 (2)	−50 ± 6 (2)	10 ± 3 (2)
HEK	Na_V_1.7 Control	−60 ± 1 (6)	8 ± 1 (6)	n/a	n/a	n/a	n/a
HEK	N‐terminal domain	−61 ± 4 (6)	8 ± 1 (6)	n/a	n/a	n/a	n/a
HEK	L1L2	−66 ± 2 (9)	9 ± 1 (9)	n/a	n/a	n/a	n/a
HEK	L3	−67 ± 3 (11)	12 ± 1 (11)	n/a	n/a	n/a	n/a
HEK	C‐terminal domain	−60 ± 2 (8)	9 ± 1 (8)	n/a	n/a	n/a	n/a

*V*_h_ and *k* value for the indicated Na_V_ currents. Values represent means ± SEM of indicated number of cells (*n*). Inactivation curves are presented in Fig. 9. *V*_h_ represents membrane potential at which inactivation is half‐maximal in cells with monophasic inactivation and *k* represents the slope factor. In cells with biphasic inactivation, *V*_h1_ and *V*_h2_ represent the membrane potential at which inactivation is half‐maximal for the current components inactivating at negative and positive membrane potentials, respectively. *k*_1_ and *k*_2_ represent the respective slope factors for the currents inactivating with *V*_h1_ and *V*_h2_. See Fig. 1 for generation of constructs.

### Biphasic inactivation of endogenous Na_V_ currents in Ins1 cells

**Figure 10:**
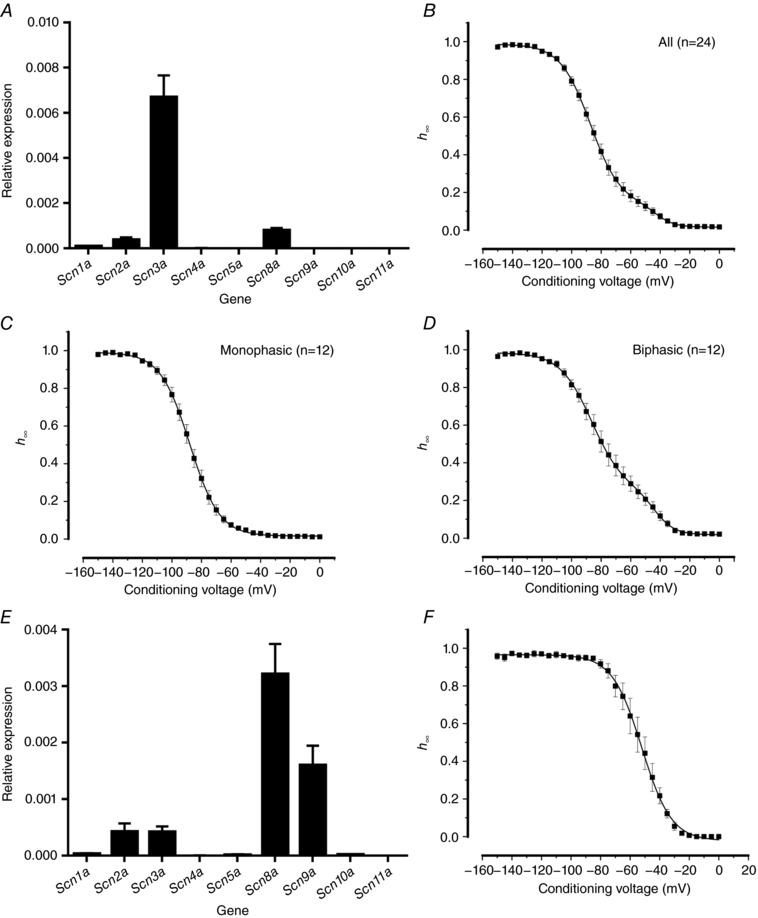
Characterization of endogenous Na_V_ currents in Ins1 and αTC1‐6 cells *A*, relative mRNA expression of Na_V_ channel α‐subunits in Ins1 cells (*n = *2 preparations). *B*, voltage dependence of inactivation of endogenous Ins1 Na_V_ currents, average of all cells (*n = *24). Curve represents a double Boltzmann fit to the data. *C*, as in *B* but for cells with Na_V_ inactivation best fitted to a single Boltzmann function (i.e. had a monophasic inactivation; *n = *12). *D*, as in *C* but for cells where inactivation was best fitted to a double Boltzmann function (i.e. had a biphasic inactivation; *n* = 12). *E*, relative mRNA expression of Na_V_ channel α‐subunits in αTC1‐6 cells (*n = *3 preparations). *F*, voltage dependence of inactivation of endogenous αTC1‐6 Na_V_ currents (*n* = 6).

We examined the expression of Na_V_ channel α­subunits in Ins1 cells and found that these cells express high levels of *Scn3a* (Na_V_1.3) and low levels of *Scn2a* (Na_V_1.2) and *Scn8a* (Na_V_1.6) (Fig. 10 *A*). We measured the inactivation of the *endogenous* Na_V_ currents, and the average inactivation curve (*n* = 24) is shown in Fig. 10 *B*. We found that whereas inactivation was monophasic in 50% of the cells (12 of 24 cells) with a *V*_h_ of −88 ± 2 mV (Fig. 10 *C*), it was clearly biphasic in the remaining cells (*n* = 12; Fig. 10 *D*). In this subgroup of cells, the major component (comprising 71 ± 6%) was similar to that in cells with monophasic inactivation and had a *V*_h_ of −87 ± 3 mV. In addition, there was a smaller component that accounted for 29 ± 6% of the total with a *V*_h_ of −43 ± 2 mV. This characteristic inactivation appears to be unique to β­cells because αTC1‐6 cells, which also express Na_V_1.2, Na_V_1.3, Na_V_1.6 and Na_V_1.7, displayed only a single component of inactivation with a *V*_h_ of −55 ± 4 mV (*n* = 6; Fig. 10 *E* and *F*).

Although we could not rule out that the expression of α‐subunits other than Na_V_1.3 explains the biphasic inactivation behaviour in Ins1 cells, we think that this is unlikely given the very low expression of *Scn2a* and *Scn8a* and we instead hypothesize that Na_V_1.3 channels expressed in the same cell may undergo inactivation with different voltage dependences.

**Figure 11:**
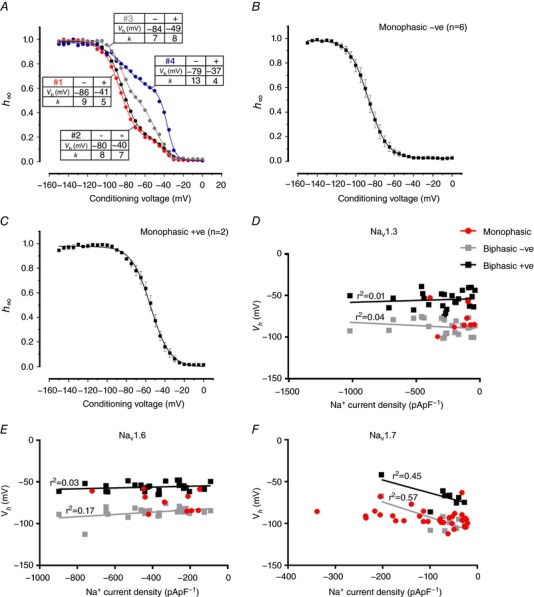
Biphasic inactivation of Na_V_1.3, 1.6 and 1.7 currents in Ins1 cells *A*, examples of biphasic inactivation of Na_V_1.3 currents when the α­subunit is co­expressed with β_1_ and β_2_ ­subunits in four different Ins1 cells (nos 1–4). The tables next to the curves show *V*_h_ and *k* values for the components inactivating at negative (−) and more positive (+) membrane potentials. The curves represent a double Boltzmann fit to the data. *B*, average voltage dependence of inactivation of Na_V_1.3 currents in Ins1 that were best described with a single Boltzmann fit to the data and had a *V*_h_ <−70 mV (*n = *6). *C*, average voltage dependence of inactivation of Na_V_1.3 current in Ins1 cells that were best described with a single Boltzmann fit to the data that had a *V*_h_ >−70 mV (*n = *2). *D*, relationship between monophasic, biphasic negative (−ve) and biphasic positive (+ve) *V*_h_ values and the peak Na^+^ current density of Na_V_1.3 in Ins1 cells. The lines represent linear regression fits to the data. The *r* ^2^ values are given next to the respective fit. *E* and *F*, same as in *D* but for Na_V_1.6 (*E*) and Na_V_1.7 (*F*). [Color figure can be viewed at http://wileyonlinelibrary.com]

Figure 11 *A* shows inactivation in four different Ins1 cells in which inactivation of expressed Na_V_1.3 channels was clearly biphasic with values of *V*_h_ ranging between −86 and −37 mV. Such cells accounted for 70% of all cells tested. In these 26 cells (of a total of 34 cells), the negative and positive components of inactivation contributed 57 ± 5% and 43 ± 5% of the total current, respectively. The corresponding values of *V*_h_ were −87 ± 2  and −55 ± 2 mV (Table 4). In addition, there were cells in which inactivation was monophasic (*n* = 8; Table 4): in most of these cells, inactivation occurred at negative voltages (*V*_h_ = −90 mV) (Fig. 11 *B*) but in two cells inactivation was instead at positive voltages (*V*_h_ = −50 mV) (Fig. 11 *C*). Similarly, in cells expressing Na_V_1.6, 25 cells out of a total of 35 cells exhibited biphasic inactivation (Table 4). For Na_V_1.7, biphasic inactivation was observed in 9 out of 47 cells (*P* < 0.001 by χ^2^ *vs*. both Na_V_1.3 and Na_V_1.6). None of the α­subunits showed biphasic inactivation when expressed in HEK, CHO or αTC1­6 cells.

**Table 4:** Monophasic and biphasic inactivation of Na_V_1.7, 1.3 and 1.6 in Ins1 cells

	Monophasic		Biphasic			
Na_V_ current	*V*_h_ (mV)	*k*	*V*_h1_ (mV)	*k*_1_	*V*_h2_ (mV)	*k*_2_
Na_V_1.3	−79 ± 6 (8)	9 ± 1 (8)	−87 ± 2 (26)	9 ± 1 (26)	−55 ± 2 (26)	7 ± 1 (26)
Na_V_1.6	−73 ± 4 (10)	8 ± 1 (10)	−86 ± 1 (25)	8 ± 1 (25)	−56 ± 1 (25)	6 ± 1 (25)
Na_V_1.7	−94 ± 2 (38)	9 ± 1 (38)	−97 ± 4 (9)	8 ± 1 (9)	−67 ± 4 (9)	10 ± 2 (9)

Same data as in Table 1, except cells are divided between those that exhibited Na_V_ current inactivation that were best described using a single Boltzmann function (monophasic) and those that were best described using a double Boltzmann function (biphasic) (Akaike information criterion test). Values represent means ± SEM of indicated number of cells (*n*). *V*_h_ represents membrane potential at which inactivation is half‐maximal in cells with monophasic inactivation and *k* represents the slope factor. In cells with biphasic inactivation, *V*_h1_ and *V*_h2_ represent the membrane potential at which inactivation is half‐maximal for the current components inactivating at negative and positive membrane potentials, respectively. *k*_1_ and *k*_2_ represent the respective slope factors for the currents inactivating with *V*_h1_ and *V*_h2_.

It is worth noticing that the fraction of cells showing biphasic Na^+^ current inactivation was not increased by substitution of cytoplasmic Na_V_1.7 for Na_V_1.3 domains (Table 3).

Figure 11 *D* shows the relationship between Na^+^ current density and *V*_h_ of the negative and positive components fitted to a linear regression with *r* ^2^ values of 0.04 and 0.01, respectively. It is clear that there is no impact of current density on *V*_h_. However, there are clearly two distinct groups that inactivate at distinct membrane potentials, separated by approximately 30 mV. Interestingly, cells that displayed monophasic inactivation fall into either of these distinct groups. Similar data were obtained for Na_V_1.6 and Na_V_1.7 channels expressed in Ins1 cells (Fig. 11 *E* and *F*).

### Modulation of inactivation

**Figure 12:**
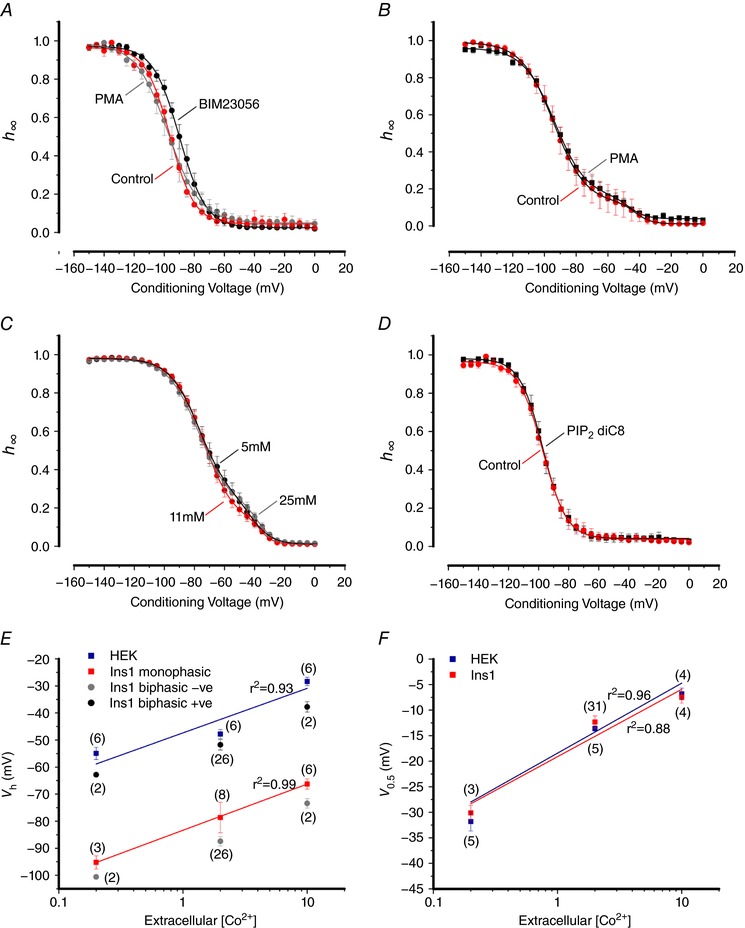
Modulation of Na_V_ inactivation in Ins1 cells *A*, voltage dependence of inactivation of Na_V_1.7 α‐subunit co‐expressed with β_1_‐ and β_2_‐subunits in control (red; *n = *5), 100 nm BIM23056 treated (black; *n = *5) and 10 nm PMA treated (grey; *n = *4) Ins1 cells. The curves represent a single Boltzmann fit to the data. *B*, voltage dependence of inactivation of endogenous Na_V_ currents in control Ins1 cells (red; *n = *4) and Ins1 cells chronically (48 h) treated with 10 nm PMA (black; *n = *11). The curves represent a double Boltzmann fit to the data. *C*, voltage dependence of inactivation of endogenous Na_V_ currents in Ins1 cells in response to a 48 h chronic incubation in 5 mm (black; *n = *22), 11 mm (red; *n = *22) and 25 mm (grey; *n = *18) glucose. *D*, same as in *A*, but for control (red; *n = *5) and 50 μm PIP_2_ diC8 treated (black; *n = *5) Ins1 cells. *E*, summarized voltage dependence of inactivation of Na_V_1.3 currents in Ins1 (red, grey and black) and HEK cells (blue) of indicated number of cells (*n*) at increasing concentrations of extracellular Co^2+^ (0.2, 2 and 10 mm). The lines represent linear regression fits to the data. The *r* ^2^ values are given next to the respective fit. *F*, same as in *E* but for the voltage dependence of activation of Na_V_1.3 currents in Ins1 (red) and HEK cells (blue). [Color figure can be viewed at http://wileyonlinelibrary.com]

Protein kinase C (PKC) phosphorylation of Na_V_1.7 exhibits a depolarizing effect on the voltage dependence of inactivation in some (Tan *et al*. 2014) but not all cell types (Vijayaragavan *et al*. 2004). Moreover, Na_V_1.3 and Na_V_1.6 contain a putative PKC phosphorylation site in the same L3 region. We therefore tested whether there was acute activation or inhibition by PKC of Na_V_1.7 inactivation by application of PMA (10 nm) or the inhibitor BIM23056 (100 nm). However, neither compound affected Na_V_1.7 inactivation (Fig. 12 *A* and Table 5). Down‐regulation of PKC (by long‐term exposure to PMA) was likewise without effect on inactivation of endogenous Na_V_ currents in Ins1 cells (Fig. 12 *B*) and it did not affect the biphasic pattern of inactivation. Consistent with earlier reports in the rat insulinoma RINm5F (Rorsman *et al*. 1986), chronic exposure to PMA reduced the amplitude of the Na_V_ current (not shown).

**Table 5:** Modulation of Na_V_ inactivation in Ins1 cells

		Monophasic		Biphasic			
Na_V_ current	Treatment	*V*_h_ (mV)	*k*	*V*_h1_ (mV)	*k*_1_	*V*_h2_ (mV)	*k*_2_
Na_V_1.7	Control	−96 ± 1 (5)	8 ± 1 (5)	n/a	n/a	n/a	n/a
Na_V_1.7	100 nm BIM23056	−90 ± 2 (5)	8 ± 1 (5)	n/a	n/a	n/a	n/a
Na_V_1.7	10 nm PMA	−96 ± 4 (4)	10 ± 1 (4)	n/a	n/a	n/a	n/a
Ins1 endogenous	Control	−94 ± 6 (2)	10 ± 1 (2)	−94 ± 1 (2)	10 ± 1 (2)	−45 ± 5 (2)	4 ± 1 (2)
Ins1 endogenous	10 nm PMA (chronic)	−91 ± 2 (6)	11 ± 1 (6)	−92 ± 2 (5)	10 ± 1 (5)	−46 ± 2 (5)	5 ± 1 (5)
Ins1 endogenous	5 mm glucose	−70 ± 3 (16)	9 ± 1 (16)	−80 ± 4 (6)	9 ± 1 (6)	−41 ± 3 (6)	5 ± 1 (6)
Ins1 endogenous	11 mm glucose	−74 ± 2 (13)	9 ± 1 (13)	−73 ± 6 (9)	9 ± 1 (9)	−43 ± 4 (9)	5 ± 1 (9)
Ins1 endogenous	25 mm glucose	−72 ± 4 (7)	9 ± 1 (7)	−78 ± 2 (11)	11 ± 1 (11)	−39 ± 2 (11)	5 ± 2 (11)
Na_V_1.7	Control	−99 ± 1 (5)	7 ± 1 (5)	n/a	n/a	n/a	n/a
Na_V_1.7	50 μm PIP_2_ diC8	−97 ± 2 (5)	7 ± 1 (5)	n/a	n/a	n/a	n/a
Na_V_1.7	Control	−98 ± 3 (7)	11 ± 2 (7)	n/a	n/a	n/a	n/a
Na_V_1.7	50 μm neomycin	−98 ± 1 (7)	8 ± 1 (7)	n/a	n/a	n/a	n/a

*V*_h_ and *k* value for the indicated Na_V_ currents and treatment conditions. Values represent means ± SEM of indicated number of cells (*n*). Inactivation curves are presented in Fig. 12. *V*_h_ represents membrane potential at which inactivation is half‐maximal in cells with monophasic inactivation and *k* represents the slope factor. In cells with biphasic inactivation, *V*_h1_ and *V*_h2_ represent the membrane potential at which inactivation is half‐maximal for the current components inactivating at negative and positive membrane potentials, respectively. *k*_1_ and *k*_2_ represent the respective slope factors for the currents inactivating with *V*_h1_ and *V*_h2_.

Na_V_ current inactivation has been reported to be regulated by the intracellular ATP concentration (Zou *et al*. 2013) suggestive of metabolic regulation. We therefore tested the effect of metabolic regulation by culturing cells at 5, 11 or 25 mm glucose for 48 h. No effects on the inactivation of endogenous Na_V_ currents was observed (Fig. 12 *C*). We also tested the acute effects of elevating glucose from 1 to 20 mm on Na_V_1.3 and Na_V_1.7 currents, but no effects on inactivation were observed over 10 min in perforated patch recordings (not shown).

Insulin‐secreting cells will be exposed to high concentrations of insulin (a biologically very active molecule). We tested the potential long‐term role of insulin signalling by treating Ins1 cells expressing Na_V_1.7 with K_ATP_ channel activator diazoxide (100 μm for 48 h) or a receptor antagonist S961 (1 μm for 24 h) (Schaffer *et al*. 2008) to inhibit its release or its action, respectively. Again, no impact on Na_V_1.7 inactivation was observed.

It has been proposed that β‐cells contain higher levels of PIP_2_ than other cells and that this, via modulation of the ATP sensitivity, explains how K_ATP_ channels remain active in intact β‐cells (Baukrowitz *et al*. 1998; Shyng & Nichols, 1998). We therefore considered the possibility that differences in PIP_2_ content could underlie the differences in Na_V_1.7 inactivation in Ins1 and HEK cells but inclusion of PIP_2_ diC8 (50 μm) into the pipette solution was without effect on Na_V_1.7 inactivation (Fig. 12 *D*). Likewise, neomycin (50 μm), which would shield the negative charges of PIP_2_ (MacGregor *et al*. 2002; Bista *et al*. 2015), was without effect on Na_V_1.7 expressed in Ins1 cells (not shown). Thus, differences in PIP_2_ concentrations in Ins1 and HEK cells is unlikely to cause of the different Na_V_ current inactivation properties.

Next we tested whether there was a difference in surface charge between Ins1 and HEK cells that might influence Na_V_ channel inactivation. One major source of negative charge is due to glycosylation of extracellular Na_V_ channel domains that consist of the carbohydrate derivative *N*­acetylneuraminic acid or sialic acid (Ednie & Bennett, 2012). To explore differences in surface charge we generated inactivation curves for Na_V_1.3 in the presence of 0.2, 2 and 10 mm extracellular Co^2+^ in Ins1 and HEK cells (Bennett *et al*. 1997; Bennett, 2002). Figure 12 *E* shows the relationship between the extracellular Co^2+^ concentration and *V*_h_ in Ins1 and HEK cells. However, increasing the Co^2+^ concentration produces the same ∼25 mV shift in Ins1 and HEK cells but the difference between the two cells persists (Fig. 12 *E*). It is also evident that both the positive and negative components of Na_V_1.3 inactivation in Ins1 cells are shifted by ∼25 mV and that *V*_h_ values for the positive components superimpose on those obtained in HEK cells for all Co^2+^ concentrations. Collectively, these data suggest that it is unlikely that differences in extracellular surface charges account for the negative Na_V_ current inactivation in Ins1 cells. We also analysed the impact of increasing Co^2+^ on the voltage dependence of activation (*V*_0.5_) of Na_V_1.3 currents. Notably, there is no difference in the *V*_0.5_ values at any of the Co^2+^ concentrations and there was a ∼20 mV shift between 0.2 and 10 mm Co^2+^.

### Biphasic inactivation of Na_V_1.3 in primary β‐cells

**Figure 13:**
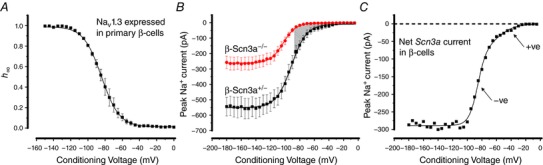
Biphasic inactivation in mouse β‐cells *A*, Na^+^ current inactivation in primary mouse β‐cells transfected with TTX­resistant Na_V_1.3 (*n = *3). *B*, voltage dependence of Na^+^ current inactivation in *Scn3a* ^+/–^ (*n = *7) and *Scn3a* ^–/–^ β‐cells (*n* = 8). Same data as in Zhang *et al*. (2014) but current amplitudes are presented in absolute rather than relative terms. *C*, net Na_V_1.3 current isolated by subtracting Na_V_ currents in *Scn3a* ^–/–^ β‐cells from those in *Scn3a^+^* ^/–^ β‐cells. The dashed line represents the zero‐current level. Note biphasic inactivation. A double Boltzmann function was fitted to the data points, yielding *V*_h_ values of −86 and −57 mV. [Color figure can be viewed at http://wileyonlinelibrary.com]

Finally we expressed Na_V_1.3 in primary mouse β‐cells. Although the transfection rate was low, large TTX‐resistant Na_V_1.3 currents were observed in three β‐cells (Fig. 13 *A*). The average current amplitude was −1 ± 0.1 nA. These currents underwent voltage‐dependent inactivation with a *V*_h_ of −88 mV. However, in two of the three cells, there was biphasic inactivation with a small (43% and 22%) component that inactivated at −95 and −103 mV and a larger (57% and 78%) component that inactivated at −73 mV in both cells.

We have previously reported that ablation of *Scn3a* leads to the loss of Na_V_ current components that inactivate at positive membrane potentials (Zhang *et al*. 2014). We have now reanalysed these data and expressed current amplitudes in absolute (in pA) rather than relative (normalized to maximum current) terms. Figure 13 *B* shows Na_V_ currents recorded in control (*Scn3a^+/−^*) and knockout *Scn3a^−/−^* β‐cells. In agreement with the previous conclusion, ablation of *Scn3a* was associated with the loss of the Na_V_ current inactivating at positive membrane potentials (Fig. 13 *B*, shaded area). However, there was also a large reduction (∼200 pA) of Na_V_ current at negative membrane potentials. Figure 13 *C* shows the net Na_V_1.3 current isolated by subtracting the currents in *Scn3a* ^−/−^ from those measured in *Scn3a* ^+/−^ mice. The net current shows biphasic inactivation and can be described as the sum of two Boltzmann functions with values of *V*_h_ of −86 and −57 mV that comprised 76% and 24%, respectively. These values are close to those observed for the isolated Na_V_1.3 current and similar to the two components of inactivation observed in Ins1 cells expressing Na_V_1.3 (Table 4).

## Discussion

We have compared the inactivation of Na_V_ channels in insulin‐secreting Ins1 and three other non‐β‐cell types (HEK, CHO and αTC1‐6 cells). To this end, we generated mutant TTX‐resistant Na_V_ channels, which allowed us to isolate the expressed channels by blocking the endogenous Na_V_ channels with TTX.

### Na_V_ channels inactivate at negative membrane potentials in Ins1 cells

We showed that Na_V_ channels inactivate at ∼30 mV more negative membrane potentials when expressed in Ins1 cells compared to what is seen in HEK cells and dorsal root ganglion neurones (Herzog *et al*. 2003; Eberhardt *et al*. 2014). We also confirmed that inactivation of Na_V_1.7 occurs at more negative membrane potentials than Na_V_1.3, Na_V_1.5 and Na_V_1.6 when these channels are expressed in Ins1 cells. Thus, although the Na_V_ α‐subunit expressed makes a significant difference to voltage dependence of inactivation, it appears that there is something special about Ins1 cells (echoing what is observed in primary β‐cells) that shifts inactivation to functionally irrelevant membrane potentials.

### Individual Na_V_ subtypes exhibit two distinct inactivation behaviours

In >75% of the experiments with Na_V_1.3, two distinct components of inactivation (each accounting for – on average – ∼50% of the total current) separated by ∼30 mV were observed. Biphasic inactivation was also observed for Na_V_1.7 (in 20% of experiments) and Na_V_1.6 (60%). Importantly, biphasic inactivation was not observed when these channels were instead expressed in HEK, CHO or αTC1­6 cells. It should also be noted that the voltage dependence for the component inactivating at more depolarized membrane potentials is very similar to that found in HEK or CHO cells (in which biphasic inactivation was never observed). In cells that showed monophasic inactivation, it proceeded at either positive *or* negative voltages but not in between. Likewise, when the measurements were done in cell‐attached patches, inactivation again tended to proceed at either negative or positive voltages but was never biphasic.

It appears that the data obtained in Ins1 cells can be extended to primary β‐cells and the net current that can be isolated by subtracting the currents recorded from the Na_V_1.3‐deficient cells from control cells likewise exhibited a biphasic voltage dependence of inactivation. Thus, it appears that although different α­subunits contribute to the biphasic inactivation, there is also an additional cell­specific modulation whereby a single α­subunit may exhibit two distinct voltage dependences of inactivation.

### Negative inactivation does not result from a diffusible factor

We explored the possibility that inactivation at negative membrane potentials reflects the interaction between the Na_V_ α­subunits and an intracellular diffusible factor. This was done by recording macroscopic Na_V_ currents in membrane patches before and after patch excision, the rationale being that wash‐out of any such factor would be more efficient in the inside‐out configuration than in the whole‐cell configuration. If such an intracellular factor shifting the inactivation of Na_V_ currents towards a more negative membrane potential exists in β‐cells, then we would expect patch excision to be associated with a change in inactivation towards more positive voltages. However, such a jump was not observed. By contrast, in both Ins1 and CHO cells for Na_V_1.3 and Na_V_1.7 channels alike, patch excision resulted in a time‐dependent shift of inactivation towards more negative membrane potentials. Why this negative shift occurs is not immediately clear but has been observed by others and might reflect the time‐dependent loss of charged molecules that affect the transmembrane voltage sensed by the inactivation particle(s) (Cachelin *et al*. 1983; Jo & Bean, 2014). However, in the context of the current study, the key observation here is the absence of even a transient shift towards more depolarized membrane potentials. Thus, we argue that there is no diffusible modulator of channel modulation. The fact that substituting the cytoplasmic domains of Na_V_1.7 for Na_V_1.3 is also without effect on the voltage dependence of inactivation also suggests that the chief determinant of Na_V_ current inactivation lies within the transmembrane domains.

We can discount the possibility that β­subunits account for the negative inactivation of Na_V_ in insulin‐secreting cells as (1) Ins1 cells and primary mouse β­cells express different β‐subunits (β_1_ and β_3_, respectively) and yet show the same negative inactivation in both cell types; (2) expressing Na_V_ with β_3_ instead of β_1_ and β_2_ has only a small (∼5 mV) effects on channel inactivation; and (3) knockdown of β‐subunits in Ins1 cells yielded the same effect on inactivation as control, suggesting that the β‐subunit is not modulated in a cell‐specific manner to modulate channel gating.

### Biphasic inactivation in Ins1 cells: possible interpretation of the data

An explanation of these data would have to account both for the fact that inactivation of all studied Na_V_ subtypes is shifted by 20–30 mV in Ins1 cells relative to that seen in other cell types (HEK, CHO, etc.) and the finding that biphasic Na^+^ current inactivation is observed also when a single Na_V_ subtype is expressed.

We considered the possibility that inactivation of the Na_V_ currents is more negative in Ins1 cells than in HEK cells as a result of surface charge effects due to differential sialylation of the channels and/or membrane. We tested this using increasing concentrations of extracellular Co^2+^. Hypothetically, Na_V_ channels expressed in Ins1 cells might carry more negative charges than channels expressed in HEK cells (for example due to increased sialylation of the extracellular domains). Accordingly, the voltage difference sensed by the inactivation gate would be reduced and this might explain the shift in inactivation towards more negative voltages. If this were the case, then increasing the divalent cationic strength would be expected to produce a greater shift in Ins1 cells (because there are more negative charges to shield). However, we think that this possibility can be discarded because varying the extracellular cationic strength had the same effects in both cell types and both negative and positive components of inactivation in Ins1 cells. Moreover, the voltage dependence of activation was superimposable in Ins1 and HEK cells and was invariably monophasic in both cell types. For both inactivation and activation, there was the same 20–25 mV shift in gating for a 50‐fold increase in extracellular Co^2+^. Altogether, these observations make it less likely that the widely different voltage dependences of inactivation in Ins1 and HEK cells can be attributed to variable surface charge effects.

**Figure 14:**
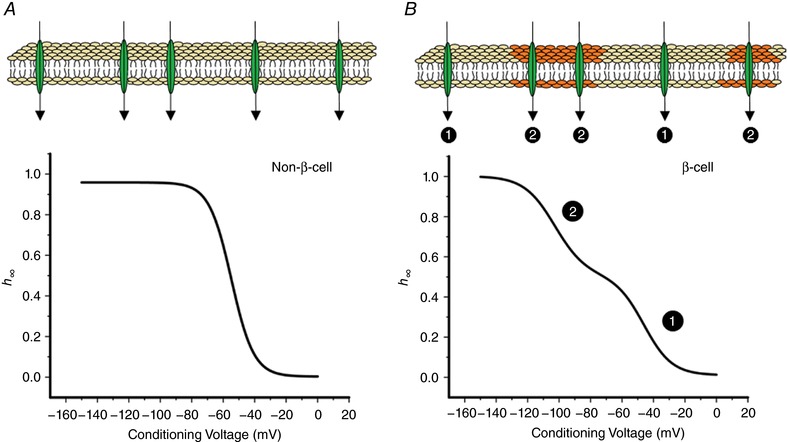
Biphasic inactivation in Ins1 cells Schematic representation of Na_V_ channel inactivation in non‐β‐cells (*A*) and β‐cells (*B*). In non­β­cells, the plasma membrane is uniform in terms of impact on Na_V_ current inactivation and the relationship between membrane potential and the fraction of activatable Na_V_ channels (*h*_∞_). However, β‐cells contain specialized domains that differ from the rest of the plasma membrane. Na_V_ channels within these domains undergo inactivation at more negative membrane potentials whereas Na_V_ channels outside these regions inactivate with the same voltage dependence as the corresponding Na_V_ channels when expressed in non‐β‐cells. Thus, the Ins1 cell will contain two electrophysiologically distinct populations of Na_V_ channels (❶ and ❷), accounting for the biphasic Na_V_ current inactivation. [Color figure can be viewed at http://wileyonlinelibrary.com]

Rather, we favour the idea that the differences between Ins1 and other cell types is likely to reside within the plasma membrane itself and does not involve cytoplasmic factors. The plasma membrane lipidome is extremely complex and contains >2000 lipid subtypes (Galbiati *et al*. 2001; Simons & Ehehalt, 2002). The heterogeneous distribution of lipids within the plasma membrane is not uniform but shows considerable regional differences. Indeed, the plasma membrane has been described as a ‘patchwork of different lipid environments’ (Edidin, 1993). Perhaps the best known example of such lipid aggregates is the lipid rafts that are enriched in cholesterol. There are many examples of lipid microdomains and cholesterol affecting ion channel function (Dart, 2010). For example, in β‐cells, cholesterol depletion causes a hyperpolarizing shift in the inactivation curve of K_V_2.1 channels (Xia *et al*. 2004). We therefore propose that the lipidome of the β‐cell plasma membrane is different from that of HEK and most other cells and that the β‐cell membrane contains specialized lipid domains that shift inactivation into very negative membrane potentials. Na^+^ channels outside these domains behave exactly as in other cell types. Indeed, in cells showing biphasic Na_V_ current inactivation, the positive component shows essentially the same voltage dependence as in non‐β‐cells (see schematic representation in Fig. 14).

The true nature of these specialized domains remains to be established but that such domains exist is suggested by the cell‐attached measurements in which a small part of the cell (1 μm^2^) is isolated and where channel inactivation was invariably monophasic but with *V*_h_ values varying between −80 and −55 mV. We speculate that this is because all channels within an individual patch reside in a uniform membrane environment, which may differ between patches. It is interesting that the ‘inactivation curve’ for Na_V_1.3 channels reconstructed from the cumulative distribution of *V*_h_ in cell‐attached patches was biphasic (with *V*_h_ values of −75 and −55 mV) and resembled that which can be recorded from individual cells expressing this α­subunit. For Na_V_1.7, the cumulative distribution was monophasic with a *V*_h_ of ∼−90 mV. Notably, 10% of the patches had *V*_h_ values ∼30 mV more positive. This is in agreement with the whole‐cell data indicating biphasic inactivation in <20% of the cells expressing Na_V_1.7. The idea that negative and positive inactivation results from insertion of the α‐subunits into membrane domains of different composition might seem in conflict with the observation that the fraction of Na_V_ channels inactivating at negative and positive membrane potentials varied for Na_V_1.3, Na_V_1.6 and Na_V_1.7. However, it is possible that the intracellular trafficking and the association of Na_V_ channels with distinct membrane domains is α‐subunit‐dependent.

The idea that Na_V_ channel inactivation is influenced by the lipid composition of the plasma membrane also raises the interesting possibility that differences in diet may explain why the same Na_V_ α­subunit inactivates at widely different voltages in rodent, dog, pig and human β‐cells (Rorsman & Ashcroft, 2018).

### Harnessing the properties of Na_V_ channels in β‐cells for therapeutic use?

It is clear that inactivation of Na_V_ channels in rodent β‐cells is special in that it proceeds at membrane potentials 20–30 mV more negative than in other cell types. In the case of Na_V_1.7 channels, this effect may be particularly dramatic in as far as all channels will have undergone inactivation at the physiological membrane potentials (−70 mV and above). If the negative shift of inactivation in β‐cells could be harnessed (i.e. by topical/local application of agents modifying the membrane), it may provide a novel means of regulating Na^+^ channel activity. Na_V_1.7 channels play an important role in pain perception (Cox *et al*. 2006; Dib‐Hajj *et al*. 2013). If the inactivation of the Na_V_1.7 channels in nociceptive neurons became more like in β‐cells, then the channels would inactivate and thus be unavailable for action potential propagation, which may represent a novel means of pain relief, especially in disease states associated with Na_V_1.7 hyperexcitability (Dib‐Hajj *et al*. 2008).

## Additional information

### Competing interests

The authors declare that they have no conflict of interest.

### Author contributions

M.G. performed all electrophysiological experiments and data analysis, except those with the *Scn3a* KO mice, which were performed by Q.Z. M.V.C. performed all molecular biology manipulations with the channels. M.G., M.V.C. and P.R. designed the experiments and interpreted the data. M.G., M.V.C. and P.R. wrote the paper. All authors have read and approved the final version of this manuscript and agree to be accountable for all aspects of the work in ensuring that questions related to the accuracy or integrity of any part of the work are appropriately investigated and resolved. All persons designated as authors qualify for authorship, and all those who qualify for authorship are listed.

### Funding

Financial support was provided by a Wellcome Senior Investigator Award to P.R. M.G. was supported by an RDM Scholars DPhil studentship. Q.Z. is a Diabetes UK RD Lawrence Fellow.
